# P-335. Acceptance of HIV couple counseling and transmission outcomes among pregnant women and their partners

**DOI:** 10.1093/ofid/ofaf695.554

**Published:** 2026-01-11

**Authors:** Thana Khawcharoenporn, Supapen Lertvutivivat, Dareena Yamoh

**Affiliations:** Thammasat University, Bangkok, Krung Thep, Thailand; Thammasat University, Bangkok, Krung Thep, Thailand; Thammasat University, Bangkok, Krung Thep, Thailand

## Abstract

**Background:**

HIV couple counseling (HIVCC) during antenatal care (ANC) can effectively reduce the risk of HIV vertical and horizontal transmission. However, HIVCC acceptance and associated factors as well as transmission outcomes during pregnancy have not been evaluated.

**Methods:**

A prospective study was conducted among pregnant women and their partners at Thammasat University Hospital. Data were collected at their first ANC using a questionnaire and included demographics, general health and obstetric history, HIV knowledge, risks and risk perception, and HIVCC acceptance. All pregnant women were prospectively followed for their HIV and syphilis outcomes until a gestational age of 32 weeks.

**Results:**

Of the 139 participating couples, the median age were 30 years for pregnant women and 32 years for their partners, most were Thai couples (59%) and 44% were in their first pregnancy. Most couples had good knowledge about HIV transmission prevention during pregnancy ( >90%). According to the study’s HIV risk categorization tool, 5% and 4% of pregnant women and her partners were categorized as moderate to high risk for HIV acquisition, and 5% of both group had false perception of low HIV risk. The HIVCC acceptance rate was 85%. The most common reasons for not accepting HIVCC were inability to come for follow-up blood tests and not wanting their partners to know the test results. Independent factors associated with no HIVCC acceptance in pregnant women were lower knowledge score [adjusted odds ratio (aOR) 2.56; *P*=0.003], self-payment for medical expense (aOR 9.81; *P*=0.008) and non-Thai nationality (aOR 3.60; *P*=0.018), while lower knowledge score (aOR 27.78; *P*< 0.001) and self-pay for medical expense (aOR 381.37; *P*=0.002) were independent factors associated with no HIVCC acceptance in their partners. There was no HIV and syphilis acquisition among all pregnant women during the follow-up period.

**Conclusion:**

The interventions to improve HIVCC in our setting should include providing pregnant women and their partners education about HIV and transmission prevention, while among those who had no coverage for their medical expenses and were non-Thai couples, strategies to reduce their financial burden should be considered.

**Disclosures:**

All Authors: No reported disclosures

